# Schooling and career aspirations of South Asian students

**DOI:** 10.1186/2193-1801-3-S1-O1

**Published:** 2014-12-04

**Authors:** Stella Sin-tung Kwok, Catherine Chiu, Brenda Lo, Richard Wu

**Affiliations:** Research Support Unit, Vocational Training Council, Kragujevac, Hong Kong; Department of General Education, Technological and Higher Education Institute of Hong Kong, Kragujevac, Hong Kong; Principal’s Office, Youth College (Tseung Kwan O), Kragujevac, Hong Kong; Department of Information & Communications Technology, Hong Kong Institute of Vocational Education (Tsing Yi), Kragujevac, Hong Kong

## Background

Numerous studies have been conducted in the West on the educational needs of minority students. These studies indicate that students from particular ethnic backgrounds have unsatisfactory educational experiences and various learning difficulties [1, 2]. Although the HKSAR government, through its equal opportunity legislation, has committed itself to eliminating ethnic inequality, the South Asian minorities are confronted with various difficulties. Recent studies on the needs of these South Asian students suggest that they face various difficulties in adjusting to living in Hong Kong due to a number of interrelated factors, including insufficient Chinese language skills, lack of a tailor-made Chinese curriculum for non-Chinese-speaking students, lack of knowledge about or access to available services, difficulties in finding school placements and employment opportunities, and discriminatory treatment [3]. Although many local studies have been conducted to investigate ethnic minority students’ difficulties in learning Chinese [4, 5], little is known about their aspirations and perceived choices in schooling, training and employment.

## Methods

This paper investigates South Asian students’ learning experiences and addresses issues of further studies and career preparation through understanding of their educational and vocational aspirations. Semi-structured interviews were carried out with Secondary 4 and 5 South Asian students in one designated and one non-designated school during April and May 2013.

## Results

The results reveal the diversity of needs among South Asian students during learning. The majority of the South Asian students interviewed aspired to ‘mainstream’ education and occupations, but they were acutely aware of their inability to fulfill their ambitions. Students’ emphasis on making themselves work harder in the pursuit of their aspirations testifies to the entrenched ideology of competitive individualism in local society. Because a limited number of affordable educational services target South Asian students at senior secondary level in the community, this calls for increased investment of the government on after school educational services for South Asian students across all levels of schooling. However, some interviewees were not aware of other educational choices except for mainstream secondary schools and universities.

## Conclusions

Educators should recognize that academic education is not the best for everyone. There are alternative education programs available for those who do not cope well in the mainstream educational path, and vocational education and training (VET) is a way to improve students’ employability. Schools serving ethnic minority students are recommended to strengthen their career guidance services and make information and knowledge of VET more available to students and parents. These include technical and vocational programs targeting Secondary 3 and Secondary 6 transitions.
